# A cell-based ribozyme reporter system employing a chromosomally-integrated 5′ exonuclease gene

**DOI:** 10.1186/s12860-021-00357-7

**Published:** 2021-03-16

**Authors:** Aiyada Aroonsri, Jindaporn Kongsee, Jeremy David Gunawan, Daniel Abidin Aubry, Philip James Shaw

**Affiliations:** 1 National Center for Genetic Engineering and Biotechnology (BIOTEC), National Science and Technology Development Agency (NSTDA), Pathum Thani, 12120 Thailand; 2 School of Life Science, Indonesia International Institute for Life Sciences (i3L), Jakarta, 13210 Indonesia

**Keywords:** Ribozyme, *E. coli* cell-based system, *glmS* riboswitch, Hammer-head ribozyme, RNaseJ1, Reporter system

## Abstract

**Background:**

Bioinformatic genome surveys indicate that self-cleaving ribonucleic acids (ribozymes) appear to be widespread among all domains of life, although the functions of only a small number have been validated by biochemical methods. Alternatively, cell-based reporter gene assays can be used to validate ribozyme function. However, reporter activity can be confounded by phenomena unrelated to ribozyme-mediated cleavage of RNA.

**Results:**

We established a ribozyme reporter system in *Escherichia coli* in which a significant reduction of reporter activity is manifest when an active ribozyme sequence is fused to the reporter gene and the expression of a foreign *Bacillus subtilis* RNaseJ1 5′ exonuclease is induced from a chromosomally-integrated gene in the same cell.

**Conclusions:**

The reporter system could be useful for validating ribozyme function in candidate sequences identified from bioinformatics.

**Supplementary Information:**

The online version contains supplementary material available at 10.1186/s12860-021-00357-7.

## Background

The molecular functions of ribonucleic acid (RNA) are diverse and essential in all organisms. Distinct classes of RNA catalyse biochemical reactions (ribozymes), although the large subunit rRNA and RNaseP ribozymes common to all organisms require protein partners to function [1]. Nucleolytic ribozymes (hereafter referred to as ribozymes for short) are small RNAs (less than 200 nt) that function independently of proteins, of which nine classes are known to exist (hammerhead, hairpin, Varkud satellite, hepatitis delta virus (HDV), *glmS*, twister, twister-sister, pistol and hatchet) each with distinctive folding patterns. They catalyze site-specific cleavage of RNA, and in some cases, the reverse ligation reaction via a concerted general acid–base mechanism [2]. Ribozymes were first discovered in viral-like RNA pathogens of plants (hammerhead and hairpin) or of humans (HDV), and sporadically in metazoan genomes [3]. More recently, different reports confirmed the widespread occurrence of HDV [4] and hammerhead [5–7] ribozymes from prokaryotes to higher metazoans, including humans [8, 9]. Besides HDV and hammerhead, other classes of ribozyme may also be widespread, including the twister ribozyme [10].

Ribozymes are modular and functional in different RNA contexts. For this reason, ribozymes can be exploited as tools to control gene expression and other applications, such as molecular sensors [11, 12]. External stimuli such as pH, temperature and cations are known to modulate the activities of naturally occurring ribozymes [12]; however, the *glmS* ribozyme is the only known example of a natural ribozyme that functions to regulate gene expression in a ligand-dependent manner by requiring glucosamine-6-phosphate (GlcN6P) as a cofactor [13]. In order to exploit other ribozymes as tools which can be controlled in a ligand-dependent manner, they must be joined artificially to a ligand-binding RNA module as aptazymes [14]. The performance of aptazymes is rather modest in vivo compared with the *glmS* ribozyme, probably because of the slow kinetics of conformation change needed for aptazyme cleavage [15]. Other classes of natural ribozymes may exist among the many candidate genomic regions identified by bioinformatics, but validation of ribozyme activity is lacking [16]. In order to validate ribozymes, in vitro assays can be used to assess RNA cleavage. These methods require extensive optimization and skill, and are difficult to conduct on a large scale. For example, four out of 18 putative hammerhead ribozymes identified by bioinformatic prediction could not verified by in vitro cleavage assay [7]. It is possible to isolate novel ribozymes encoded in a genome by a high-throughput, iterative in vitro ribozyme assay. However, this method is limited to ribozymes less than 150 nt in length and up to 12 laborious enrichment cycles are needed to isolate ribozymes [8]. The length limitation for this technique could prevent the isolation of novel ribozymes that may reside among the numerous long non-coding RNAs (greater than 200 nt) encoded in bacterial genomes [17].

In addition to in vitro ribozyme assays, ribozyme function can be tested by in vivo reporter assays. In these assays, the ribozyme sequence is fused to a reporter gene which modulates its activity when expressed as RNA. In vivo reporter assays are most commonly used to select candidate ribozymes or aptazymes for in vivo application for which some information of ribozyme activity is known from in vitro assays. In the simplest reporter systems, loss of reporter activity is correlated with ribozyme activity. These systems can be used to verify mutations in sites known to be important for ribozyme cleavage, or screen for small molecule inhibitors of ribozyme [18]. More elaborate reporters can be used to select for active ribozymes among a pool of variants [19], or test novel aptazyme designs using combinations of ribozyme and ligand-binding aptamer [20]. Current in vivo reporter systems cannot be used to validate the activity of a novel ribozyme because the effect on reporter activity is highly dependent on the sequence context, and does not correlate with ribozyme activity in vitro [21]. Ribozyme activity can be demonstrated more reliably in vivo using reporters expressed in different cell backgrounds, including mutants of mRNA processing in yeast [22] and *Bacillus subtilis* Gram-positive bacteria [23]. In this approach, ribozyme-cleaved reporter RNA is more stable in mutants, leading to higher expression. As most mRNA processing genes are important for viability, loss of function mutants have growth and morphological defects [24, 25], which can confound reporter assays where vigorous growth is required for reporter expression.

All ribozymes self-cleave to produce one fragment with a 2′,3′-cyclic phosphate terminus and another with a 5′-OH [2]. In *Escherichia coli* and other Gram-negative bacteria, RNA with 5′-monophosphate is degraded by the RNaseE system, whereas RNAs with 5′-OH termini are stable because these organisms lack a 5′ exonuclease [26]. In contrast, ribozyme-cleaved reporter RNA is rapidly degraded by the 5′ exonuclease RNaseJ1 in Gram-positive bacteria, as shown for the *glmS* and hammerhead ribozyme products [23]. In the same study, it was shown that ribozyme-cleaved reporter RNA with a 5′-OH is degraded in *E. coli* only upon expression of foreign *B. subtilis* RNaseJ1 [23] from a separate plasmid. These data establish the basis for a simple in vivo reporter assay of ribozyme function, in which ribozyme-mediated cleavage of reporter RNA only leads to loss of reporter protein upon induction of RNaseJ1. However, it was not shown whether measurement of reporter protein from ribozyme-cleaved reporter RNA in *E. coli* is sufficiently robust to demonstrate ribozyme activity. We hypothesized that a fluorescent protein reporter could be used to demonstrate ribozyme activity in *E. coli* expressing *B. subtilis* RNaseJ1. To test this hypothesis, we established *E. coli* transgenic lines expressing RNaseJ1 under inducible control and a reporter gene fused to ribozyme sequences. We established conditions in which a significant change in reporter activity was only observed when active ribozyme was fused to the reporter gene and cells expressed reporters and RNaseJ1 from chromosomally-integrated genes. The reporter system could be used to assess ribozyme activity, including that of novel ribozymes.

## Results

**Fig. 1:**
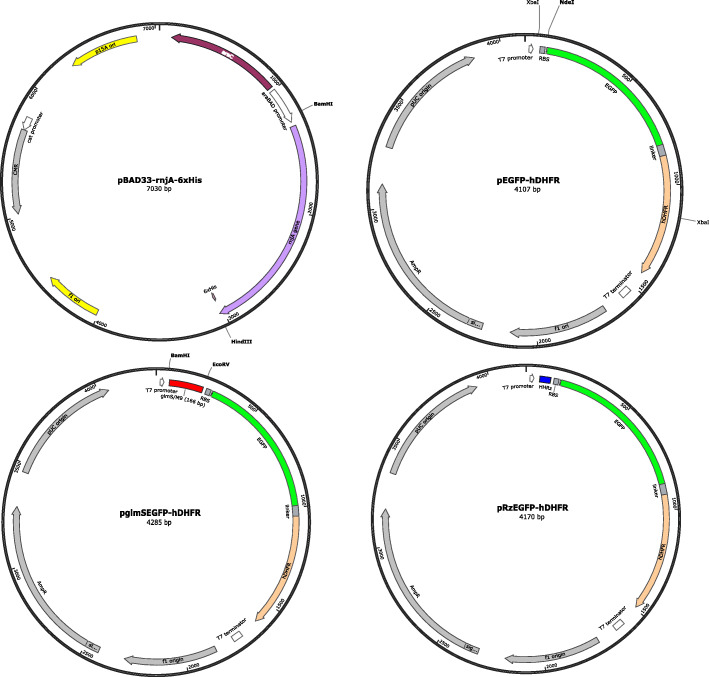
Schematic of pBAD33-rnjA-6xHis plasmid and reporter plasmids pEGFP-hDHFR, p*glmS*EGFP-hDHFR and pRzEGFP-hDHFR. Plasmid maps were drawn using SnapGene® Viewer software version 5.1**.**3.1 (Insightful Science, [27])

**Fig. 2:**
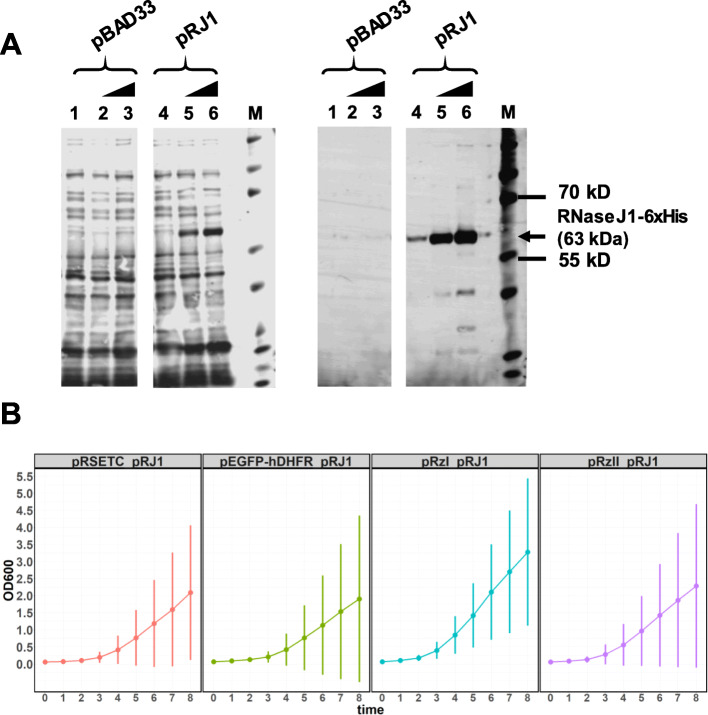
Phenotypic analysis of plasmid transformants. **a** Western immunoblot analysis to detect RNaseJ1-6xHis in *E. coli* DH5α harboring pBAD33 (pBAD33, lane 1–3) and pBAD33-rnjA-6xHis (pRJ1, lane 4–6) cultured in 0, 0.0012, and 0.0037% (w/v) arabinose as shown by the wedge above the lanes. Total protein-stained membrane is shown in the left panel and immunodetection of RNaseJ1-6xHis using Anti-6X His IgG, CF™680 (Sigma-Aldrich, Merck KGaA, Germany) is shown in the right panel. The expected size of RNaseJ1-6xHis is 63 kDa, as shown by the arrow. Lane M indicates KaledioscopeTM prestained protein ladder (Bio-Rad). **b** Growth analysis of *E. coli* BL21(DE3) co-transformants of pRSETC/ pBAD33-rnjA-6xHis (pRSETC_pRJ1), pEGFP-hDHFR/ pBAD33-rnjA-6xHis (pEGFP-hDHFR_pRJ1), pRzIEGFP-hDHFR/ pBAD33-rnjA-6xHis (pRzI_pRJ1), pRzIIEGFP-hDHFR/ pBAD33-rnjA-6xHis (pRzII_pRJ1). OD_600_ was measured every hour from 0 to 8 h cultivation time in cultures grown in HDAG medium with no arabinose and data were plotted using the Growthcurve package in R software. Points represent mean of 2–4 experiments and error bars represent 95% confidence intervals

We constructed plasmids for expression of reporter protein and *B. subtilis* RNaseJ1 with compatible origins that can be co-transformed into *E. coli* similar to [23]. (Fig. 1). The transformant carrying pBAD33-rnjA-6xHis (pRJ1, Fig. 1) expressed RNaseJ1 upon arabinose induction (Fig. 2a), but we observed highly variable growth among co-transformed lines, even in the absence of arabinose (Fig. 2b). From these results, we hypothesized that over-expression of RNaseJ1 from a plasmid could lead to growth inhibition. A significant arabinose dose-dependent reduction of growth was observed for *E. coli* transformed with pRJ1 but not pBAD33 control, consistent with the idea that RNaseJ1 over-expression inhibits growth (Fig. S1). The erratic growth patterns of co-transformed lines confounded accurate measurement of reporter protein for assessing ribozyme activity. No significant effect of ribozyme on reporter signal could be demonstrated owing to high variance in the reporter assays (Table S3).

**Fig. 3:**
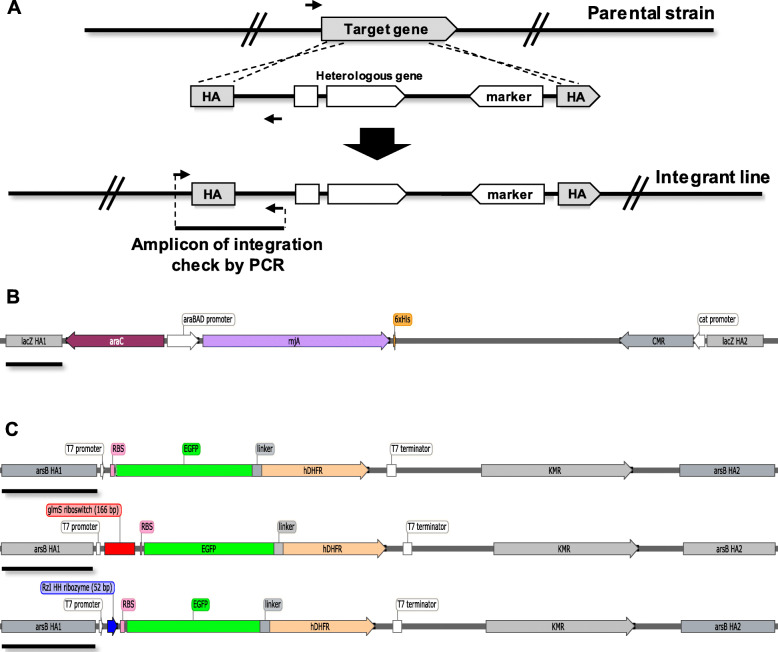
Schematic of genome integration (**a**) and DNA fragment for *E. coli* BL21(DE3) *lacZ* and *arsB* gene integration (**b** and **c**, respectively). Elements in part A are not to scale, whereas elements in B and C are drawn to scale (bar = 500 bp)

**Fig. 4:**
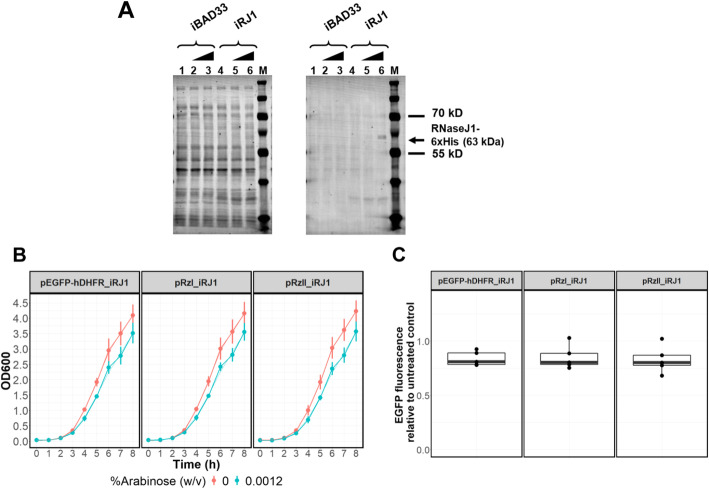
Phenotypic analysis of iRJ1 integrants harboring reporter plasmids. **a** Western immunoblot analysis to detect RNaseJ1-6xHis protein expressed in *E. coli* BL21(DE3) with integration of pBAD33-containing control DNA fragment (iBAD33, lane 1–3) and RNaseJ1-expressing gene cassette (iRJ1, lane 4–6) cultured in 0, 0.0012, and 0.0037% (w/v) arabinose as shown by the wedge above the lanes. Total protein-stained membrane is shown in the left panel and immunodetection of RNaseJ1-6xHis using Anti-6X His IgG, CF™680 (Sigma-Aldrich, Merck KGaA, Germany) is shown in the right panel. The expected size of RNaseJ1-6xHis is 63 kDa, as shown in the arrow. Lane M indicates KaledioscopeTM prestained protein ladder (Bio-Rad). **b** Growth analysis of iRJ1 integrants harboring reporter plasmids, pEGFP-hDHFR (pEGFP-hDHFR_iRJ1), pRzIEGFP-hDHFR (pRzI_iRJ1), and pRzIIEGFP-hDHFR (pRzII_iRJ1). OD_600_ was measured every hour from 0 to 8 h cultivation time and data were plotted using the Growthcurve package in R software. Data are shown for each cell type grown in the presence or absence of 0.0012% (w/v) arabinose. Points represent the mean of 5 experiments and error bars represent 95% confidence intervals. *P-*values from two sample *t*-tests for pEGFP-hDHFR_iRJ1, pRzI_iRJ1, and pRzII_iRJ1 integrants comparing growth in the presence or absence of 0.0012% (w/v) arabinose are 0.00427, 0.00523, and 0.00373, respectively. **c** Fluorescence intensity of pEGFP-hDHFR_iRJ1, pRzI_iRJ1, and pRzII_iRJ1 integrants cultured in 0.0012% (w/v) arabinose relative to untreated control. Box plots show relative fluorescence data distribution. Dots indicate relative fluorescence data from individual experiments, and median values are indicated by the bold black line. *P-*values from single value two-tailed *t*-tests comparing group mean to 1 of pEGFP-hDHFR_iRJ1, pRzI_iRJ1, and pRzII_iRJ1 are 0.005381, 0.04022, and 0.03996, respectively

To overcome the problem of high variance associated with plasmid-based gene expression, the heterologous genes were stably integrated via homologous recombination into the *E. coli* chromosome. Non-essential *E. coli* genes have been identified as integration sites for foreign genes with no effect on growth rate when disrupted [28]. We integrated the RNaseJ1 gene or control pBAD33 fragment of similar size into the *lacZ* locus (Fig. 3a and b). Integrants were identified among transformed colonies by PCR (Fig. S2). RNaseJ1 expression was observed from the RNaseJ1 gene-carrying integrant (iRJ1) upon induction with arabinose (Fig. 4a). The iRJ1 integrant transformed with different reporter plasmids exhibited consistent growth (Fig. 4b). From these results, we infer that RNaseJ1 expression is more tightly controlled when the RNaseJ1 gene is integrated, and iRJ1 cells can tolerate reporter plasmids with less effect on growth than co-transformants. We selected 0.0012% (w/v) arabinose as the inducing condition in further experiments, as this was the minimum in which RNaseJ1 protein could be detected (Fig. 4a) with a small negative effect on growth (Fig. 4b). The reporter protein signal was compared between uninduced and induced conditions. If reporter gene RNA is cleaved by the action of the ribozyme, the reporter RNA with a 5′-OH is expected to be degraded by RNaseJ1 [23] and lead to specific reduction of reporter protein. A small, but significant reduction of reporter protein was observed in iRJ1 integrant transformed with plasmid pRzIEGFP-hDHFR (pRzI_iRJ1); however, the effect was not specific as similar reductions were observed in control strains pEGFP-hDHFR_iRJ1 and pRzII_iRJ1 (Fig. 4c).

**Fig. 5:**
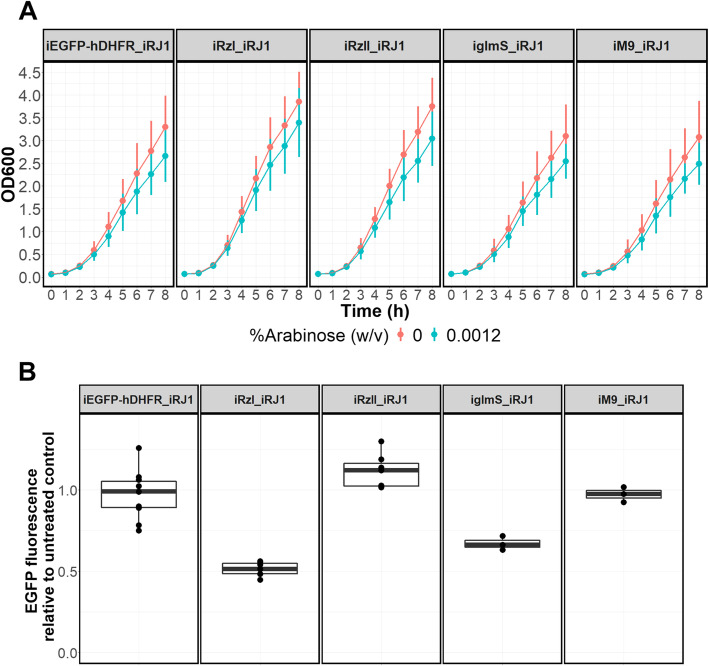
Growth analysis and fluorescence intensity of iRJ1 double integrants. **a** Growth analysis of double integrants *ΔarsB*::EGFP-hDHFR*ΔlacZ*::RJ1 (iEGFP-hDHFR_iRJ1), *ΔarsB*::RzIEGFP-hDHFR*ΔlacZ*::RJ1 (iRzI_iRJ1), *ΔarsB*::RzIIEGFP-hDHFR*ΔlacZ*::RJ1 (iRzII_iRJ1), *ΔarsB*::*glmS*EGFP-hDHFR*ΔlacZ*::RJ1 (i*glmS*_iRJ1), and *ΔarsB*::M9EGFP-hDHFR*ΔlacZ*::RJ1 (iM9_iRJ1). OD_600_ was measured every hour from 0 to 8 h cultivation time and data were plotted using the Growthcurve package in R software. Data are shown for each cell type grown in the presence or absence of 0.0012% (w/v) arabinose. Points represent the mean of 4–12 experiments and error bars represent 95% confidence intervals. *P-*values from two sample *t*-tests for iEGFP-hDHFR_iRJ1, iRzI_iRJ1, iRzII_iRJ1, i*glmS*_iRJ1, and iM9_iRJ1 integrants comparing growth in the presence or absence of 0.0012% (w/v) arabinose are 0.07435, 0.3102, 0.1096, 0.3532, and 0.345, respectively. **b** Fluorescence intensity of double integrants cultured in 0.0012% (w/v) arabinose relative to untreated control. Box plots show relative fluorescence data distribution. Dots indicate relative fluorescence data from individual experiments, and median values are indicated by the bold black line. *P-*values from single value two-tailed *t*-tests comparing group mean to 1 of iEGFP-hDHFR_iRJ1, iRzI_iRJ1, iRzII_iRJ1, i*glmS*_iRJ1, and iM9_iRJ1 are 0.5759, 0.0002, 0.0261, 0.0055, and 0.4059, respectively

Since cell growth was more consistent when RNaseJ1 was expressed from an integrated gene, we wondered if expressing reporter from integrated genes would be beneficial. We established *E. coli* double gene integrants with the RNaseJ1 gene or control pBAD33 fragment integrated at the *lacZ* locus and reporter gene cassette integrated at the *arsB* locus (Fig. 3 and S3). Double integrant cells grew robustly, with a minor reduction in the induced condition for cells with integrated RNaseJ1 (Fig. 5a). Growth of double integrant cells with integrated control pBAD33 fragment (i33) and integrated reporter was unaffected by inducer (Fig. S4A). Significant reduction of reporter protein was only observed in cells with RNaseJ1 gene and reporter protein genes with upstream active ribozyme sequences (iRzI_iRJ1 and i*glmS*_iRJ1) (Fig. 5b). In contrast, no difference in reporter signal was observed in controls, including reporter gene lacking upstream ribozyme sequence (iEGFP-hDHFR_iRJ1), reporter with upstream catalytically inactive ribozyme (iRzII_iRJ1 and iM9_iRJ1), (Fig. 5b), and cells lacking RNaseJ1 (Fig. S4B). In cells with integrated RNaseJ1 gene, the protein was produced to a similar level (Fig. S5). These results suggest that it is necessary for the reporter protein and RNaseJ1 to be expressed from integrated genes in order to detect specific signals of ribozyme activity.

## Discussion

A cell-based reporter system was established for demonstrating hammerhead and *glmS* ribozyme activity via the assessment of reporter protein production. The established *E. coli* cells contained two foreign genes encoding EGFP-hDHFR reporter protein and RNaseJ1 ribonuclease enzyme. Initially, expression of the proteins was tested in single plasmid transformant or co-transformant cells. Highly variable growth and reporter production were observed in these transformants (Fig. 2b and Table S3) that reduced power to detect ribozyme activity. Plasmids impose a metabolic burden on transformed *E. coli* owing to the expression of plasmid encoded genes and replication of plasmid DNA [29]. The overproduction of RNaseJ1 inhibits growth of the pRJ1 plasmid transformant (Fig. S1). RNaseJ1 functions as a 5′-3′ exonuclease enzyme which plays a pivotal role in *B. subtilis* RNA metabolism. Although RNaseJ1 is not present in *E. coli*, it has some functional overlap with *E. coli* RNaseE [30]. The overexpression of RNaseJ1 in *E. coli* transformants carrying pRJ1 plasmid thus might have an impact on general RNA metabolism, affecting cell homeostasis and growth.

The degree of the metabolic load in plasmid transformants is also dependent on plasmid copy number and size [29]. Poor growth of co-transformants over-expressing RNaseJ1 (Fig. 2b) could also be exacerbated by the extra burden of carrying two plasmid types lacking segregation control. The reporter gene-expressing plasmids contain a pUC origin of replication, whereas the RNaseJ1-expressing plasmid contains a p15A origin of replication (Fig. 1). Plasmid copy numbers of these two replication systems are comparable [31]. However, the transformant population is heterogeneous owing to the unequal distribution of cellular components in cell division [32, 33], which is manifest as a bimodal distribution of plasmid copy number [31]. To mitigate metabolic burden, the heterologous genes were stably integrated via homologous recombination into the *E. coli* chromosome. Previous studies have demonstrated that genome integration of foreign genes is superior to plasmid-based expression, in which the heterologous protein is expressed more stably and with less impact on growth [34–36]. In concordance with previous studies showing the benefit of gene integration, the growth of iRJ1 with integrated RNaseJ1 gene transformed with different reporter plasmids was more consistent compared with plasmid co-transformants, with only minor retardation in the induced condition (Fig. 4b). However, induction of RNaseJ1 expression in iRJ1 transformants had a small and unspecific effect on plasmid-expressed reporter signal such that it was not possible to demonstrate ribozyme activity (Fig. 4c). We do not know why ribozyme activity could not be demonstrated with plasmid-based reporters, but the combined metabolic burden of carrying reporter plasmids and expressing heterologous proteins may create a cellular condition in which RNaseJ1 is less active and/or mRNA is globally more stable.

In contrast to iRJ1 plasmid transformants, specific effects of upstream active ribozyme sequences on reporter activity were demonstrated in double integrants. However, the mean level of reporter protein for the +ara condition was less than two-fold different from the corresponding −ara condition (Fig. 5b). This suggests that the degradation of ribozyme-cleaved reporter RNA by RNaseJ1 has a modest effect on the level of reporter protein under the assay conditions used. Although degradation of ribozyme-cleaved RNA by RNaseJ1 is rapid in *E. coli* [23], the reporter protein used in our assays may be stable owing to the EGFP moiety that has a half-life of more than 24 h in *E. coli* [37]. The reporter protein signal could thus persist even after RNaseJ1-mediated mRNA degradation. Reporter protein stability can be reduced by fusing with a degron, or degradation tag for in vivo proteolysis. For example, C-terminal fusion of the ssrA peptide degron can direct fusion proteins for degradation by the endogenous ClpXP and ClpAP proteases, leading to rapid protein degradation in *E. coli* [38]. The reporter protein with a shorter half-life will be beneficial for widening the dynamic range of the reporter assay. In addition, the ribozyme activity can be monitored in real time along with bacterial growth. As an alternative to protein reporters, turn-on fluorescent RNA reporters could be used [39]. RNA reporters expressed on the same RNA molecule as the ribozyme could give a better time-resolved signal of ribozyme activity, although extensive empirical testing may be required to establish a general ribozyme reporter system. Turn-on fluorescent RNA reporters are generally less bright and thus more difficult to quantify than fluorescent proteins [39], although their performance can be improved by flanking scaffolds, which act by stabilizing the in vivo folding of the RNA reporter [40]. However, scaffolded RNA reporters are more resistant to ribonucleases [40], which may interfere with the detection of ribozyme activity as RNaseJ1-mediated loss of reporter after ribozyme cleavage.

Using the double-integrant reporter system, we demonstrated the activities of the constitutively active (RzI) and cofactor-dependent (*glmS*) ribozyme. The latter was surprising as no exogenous cofactor was added in the assay, suggesting that the intracellular level of the *glmS* cofactor, GlcN6P, is sufficiently high for in vivo ribozyme activity. The level of GlcN6P in *E. coli* varies from 0.062 to 9 mM, depending on the available carbon source [41]. This concentration range is sufficient to activate the *glmS* ribozyme in vitro [13]. In other cell types such as *Saccharomyces cerevisiae* yeast [42] and *Plasmodium falciparum* malaria parasite [43], the *glmS* ribozyme is inactive unless the intracellular GlcN6P level is increased by treatment with exogenous sugar that can be converted to GlcN6P. The *glmS* ribozyme cleaves more slowly in vitro than the hammerhead ribozyme at physiological concentrations (≈ 1 mM) of magnesium (*glmS* rate constant (K_obs_) < 1 min^− 1^, [13, 44]; hammerhead K_obs_ > 1.2 min^− 1^ [7]), which could partly explain why the reduction of reporter in the induced condition is less for i*glmS*_iRJ1 than iRzI_iRJ1 (Fig. 5b). It would be interesting to test other classes of ribozymes for further comparison in our reporter system, including the rapidly cleaving twister ribozyme (twister K_obs_ ≈ 1000 min^− 1^, [10]).

## Conclusions

An in vivo reporter system in *E. coli* was established in which ribozyme activity can be demonstrated by the specific attenuation of reporter under the condition in which RNaseJ1 expression is induced. The integration of RNaseJ1-expressing gene was necessary to ensure consistent growth and quantification of reporter signal. Specific attenuation was observed with two different types of ribozyme, suggesting that the system may be generalized to other types of ribozyme. The system could be applied for identifying novel ribozymes from candidate sequences identified by bioinformatics, e.g. among conserved non-coding RNAs. The requirement for integration of reporter gene is a limitation for high-throughput studies, although this could be circumvented by application of high-efficiency, programmable transposon integration systems [45].

## Methods

### Strains and growth conditions

*E. coli* strain DH5α and BL21(DE3) were used for recombinant DNA manipulations and heterologous expression of reporter genes, respectively. *E. coli* was cultured at 37 °C in Luria-Bertani (LB) medium [46], M9 minimal medium [46] supplemented with 0.2% casamino acids (M9CA) or Hi-def Azure (Teknova, USA) supplemented with 0.5% glycerol (HDAG). Ampicillin, chloramphenicol, and kanamycin (Sigma-Aldrich, Merck KGaA, Germany) were used at 50, 10, and 50 μg/mL final concentration, respectively.

### Plasmid construction

To construct RNaseJ1-expressing plasmid, a synthetic DNA containing the *Bacillus subtilis rnjA* gene sequence (NCBI Gene ID: 939483) with in-frame C-terminal flexible linker (GGSGGGSGG) and a six histidine residue tag (6xHis) was ordered from Genscript. The fragment was subcloned into the pBAD33 plasmid vector [47] via the BamHI and HindIII restriction sites, resulting in the pBAD33-rnjA-6xHis plasmid (Fig. 1). A reporter gene coding for a fusion protein with moieties of haemagglutinin (Ha) tag, enhanced green fluorescent protein (EGFP) [48] and human dihydrofolate reductase (hDHFR) was constructed from DNA fragments obtained as PCR amplicons. The HaEGFP fragment was PCR amplified from plasmid pcDNA5-FRT-kozak-Ha-EGFP-Halotag2 [49] using primers HaEGFP_F and HaEGFP_R (Table S1). The hDHFR gene was PCR-amplified from plasmid pL0035 [50] using primers hDHFR_F and hDHFRR4. The hDHFR amplicon was digested with KpnI and ligated to KpnI-digested HaEGFP fragment. The ligation product was used as a template for PCR using primers EGFPhDHFR_F and EGFPhDHFR_R. The amplicon was digested with BamHI and XhoI and cloned into plasmid pGFP_*glmS* [43] digested with BglII and XhoI to make pNoRbz_HaEGFP_hDHFR plasmid.

To construct reporter plasmid with ribozyme variants, the HaEGFP-hDHFR fusion gene was amplified from pNoRbz_HaEGFP_hDHFR plasmid with primers EGFP_F and hDHFRR4 (Table S1). The EGFP_F and hDHFRR4 primed amplicon was digested with NdeI and PstI enzyme and subsequently cloned into pRSETC plasmid (Invitrogen), resulting in pEGFP-hDHFR plasmid (Fig. 1). The *B. subtilis glmS* riboswitch and M9 inactive variant sequences reported in [43] were obtained by PCR. Primers BsglmS_F and 168BsglmS_R were used to amplify a 168 bp *glmS* fragment. The fragment was assembled with NdeI-digested pBAD-EviB [51] fragment using a Gibson assembly kit (New England Biolabs), resulting in E168glmS plasmid. To insert *glmS* riboswitch sequence upstream of the *egfp-hdhfr* gene, the plasmid was digested with XbaI enzyme. A 3-kb backbone vector was then assembled with two amplified fragments. The first fragment was amplified using E168*glmS* as a template and primer pBEAR166glmSgibson_F and pBEAR166glmSgibson_R for *glmS*-containing fragment or pBEAR166M9gibson_F and pBEAR166glmSgibson_R for M9-containing fragment. The second fragment was amplified using EGFP-hDHFR as a template and primer hDHFRgap_F and hDHFRgap_R. Three fragments were mixed with Gibson assembly mixture (New England Biolabs) and incubated at 50 °C for 15 min, and the mixture was then transformed into *E. coli* DH5α. The assembled plasmids were named as p*glmS*EGFP-hDHFR (Fig. 1) and pM9EGFP-hDHFR, respectively. Active (RzI) and inactive (RzII) hammerhead ribozyme sequences previously tested with reporter genes in *E. coli* [52] were obtained by PCR using oligos RzI_F + RzI_R and RzII_F + RzI_R (Table S1), respectively. The RzI sequence is a variant of the natural hammerhead sequence from the satellite RNA of tobacco ringspot virus [53, 54]. The RzII sequence has a single nucleotide substitution from RzI (C to G) at the cleavage site. The 53-bp RzI or RzII fragments were assembled with BamHI and EcoRV digested p*glmS*EGFP-hDHFR (Fig. 1) fragment plasmid to create plasmids pRzIEGFP-hDHFR and pRzIIEGFP-hDHFR, respectively (Fig. 1). The integrity of all constructed plasmids was established by Sanger DNA sequencing (Macrogen, Korea and Bioneer, Daejeon, Korea). Subsequently, the pRSETC, pEGFP-hDHFR, p*glmS*EGFP-hDHFR, pM9EGFP-hDHFR, pRzIEGFP-hDHFR and pRzIIEGFP-hDHFR plasmids were co-transformed with pBAD33 or pBAD33-rnjA-6xHis (pRJ1) plasmid into *E. coli* BL21(DE3). The co-transformants were named as pRSETC_p33, pEGFP-hDHFR_p33, p*glmS* _p33, pM9_p3, pRzI_p33, pRzII_p33, pRSETC_pRJ1, pEGFP-hDHFR_pRJ1, p*glmS*_pRJ1, pM9_pRJ1, pRzI_pRJ1 and pRzII_pRJ1, respectively. Sequences of all plasmids and DNA fragments for gene integration are provided in Supplementary data S1.

### Integration of heterologous genes in *E. coli* BL21(DE3) nonessential genes

For single integrants, the DNA fragment containing the *rnjA* gene and chloramphenicol resistance gene (CMR) was prepared for integration at the non-essential *E. coli* BL21(DE3) *lacZ* gene (NCBI ID: CAQ30819.1) encoding beta-galactosidase. 5′- and 3′- homology arms for *lacZ* integration [28] were amplified from *E. coli* BL21(DE3) genomic DNA using primer pairs HA1lacZ_F/lacZAraC_R and HA2lacZ_F/HA2lacZ_R, respectively (Table S2). Primer AraC_F and RJ1_R were used for the amplification of fragment containing *rnjA* gene and CMR or only CMR control using pBAD33-rnjA-6xHis or pBAD33 plasmid, respectively. The amplified 5′-HA, *rnjA* gene and CMR or only CMR, and 3′-HA fragments were assembled by overlap-extension PCR using primers HA1lacZ_F and HA2lacZ_R. Subsequently, the assembled fragments were cloned into EcoRV-digested and blunted pET17b plasmid fragment for sequence verification. Linearized DNA fragments (Fig. 3b) were prepared by NotI and SpeI digestion and transformed by electroporation into *E. coli* BL21(DE3) harboring pKD46, a λ-red recombinase expression plasmid [55]. λ-red mediated-recombination at *lacZ* gene resulted in *lacZ*-disrupted mutants harboring pBAD33 fragment (i33) or pBAD33-rnjA-6xHis fragment (iRJ1).

For double integrants, the *arsB* non-essential gene (NCBI ID: CAQ33821.1), which encodes ArsB arsenite transporter was chosen as a site for chromosomal integration of reporter genes in *E. coli* BL21(DE3). 5′- and 3′-homology arm (HA) fragments for *arsB* integration as described previously [28] were amplified from *E. coli* BL21(DE3) genomic DNA using primer pairs HA1arsB_F/HA1arsB_R and HA2arsB_F/HA2arsB_R, respectively (Table S2). The HAEGFP-hDHFR reporter genes and ribozyme variants were amplified using pRSETC, pEGFP-hDHFR, p*glmS*EGFP-hDHFR, pM9EGFP-hDHFR, pRzIEGFP-hDHFR, and pRzIIEGFP-hDHFR plasmids as templates and primer T7P_F and T7T_R. A kanamycin resistance gene (KMR) was used as a selectable marker in the *ΔarsB* integrants, which was amplified using pKD4 plasmid [55] as a template and primer FRT_F and FRT_R. The amplified 5′-HA, reporter genes with ribozyme variants, KMR, and 3′-HA fragments were assembled by overlap-extension PCR using primers HA1arsB_F and HA2arsB_R. Subsequently, the assembled fragments were cloned into EcoRV-digested and blunted pET17b plasmid fragment for sequence verification. Linearized DNA fragments were prepared by NotI and SpeI digestion (Fig. 3c) and transformed by electroporation into *E. coli* BL21(DE3) harboring pKD46 [55]. λ-red mediated-recombination at *arsB* gene resulted in *arsB*-disrupted mutants harboring reporter genes with different upstream regulatory elements. The integrant lines obtained are named as iRSETC, iEGFP-hDHFR, i*glmS*, iM9, iRzI, and iRzII. Next, the DNA fragment containing the *rnjA* gene and CMR or CMR control for the integration at *lacZ* gene was prepared as described above for the single integrant transformed by electroporation into *arsB*-disrupted mutants harboring pKD46. The double integrants were named as iRSETC_iRJ1, iEGFP-hDHFR_iRJ1, i*glmS*_iRJ1, iM9_iRJ1, iRzI_iRJ1, iRzII_iRJ1, iRSETC_i33, iEGFP-hDHFR_i33, i*glmS*_i33, iM9_i33, iRzI_i33, and iRzII_i33. All gene integrations were verified by PCR analysis using primers described in Table S2. Genomic DNA (gDNA) were isolated from bacterial culture using a Bacteria Genomic DNA Kit (Geneaid, Taiwan) or a Quick-DNA Miniprep kit (ZYMO RESEARCH, USA) and used for PCR amplification. 10–20 ng of gDNA was PCR amplified with Phusion polymerase following manufacturure’s recommendations (Thermo Scientific, USA).

### Growth analysis and reporter assay in *E. coli* co-transformants and integrants

Cultures of co-transformants or integrants were prepared as follows: LB medium supplemented with appropriate antibiotics was inoculated with a single colony grown overnight at 37 °C. The cells were harvested and then resuspended in fresh HDAG and appropriate antibiotics. Cell cultures were diluted to OD_600_ of 0.05 and mixed with 10 μL of arabinose solution or 1X phosphate buffered saline (PBS, 137 mM NaCl, 2.7 mM KCl, 8 mM Na_2_HPO_4_, 2 mM KH_2_PO_4_, pH 7.4) in a 96 well plate to obtain 200 μL culture volume. The cultures were incubated at 37 °C with shaking at 800 rpm. Bacterial growth was measured by OD_600_ spectrometry. Fluorescence of EGFP protein were detected by using Synergy™ Mx Fluorescence Microplate Reader (BioTek®) with excitation wavelength at 488 nm and emission wavelength at 530 nm. The fluorescence intensities were detected after 6 h cultivation time and normalized to cell turbidities (OD_600_).

### Western analysis of RNaseJ1-6xHis

Equal numbers of cells from each treatment condition were taken for protein extraction (estimated from the OD_600_ absorbance). Cells were harvested and mixed with NuPAGE® LDS sample buffer (Thermo Scientific, USA) to extract proteins. After heating at 90 °C for 5 min, protein samples were loaded onto NuPAGE 4–12% Bis-Tris Protein Gel (Thermo Scientific, USA) and separated by electrophoresis at 170 V for 50 min. Subsequently, proteins were transferred onto Immobilon-FL PVDF membrane (Merck KGaA, Germany) at 20 V for 60 min. Total proteins were stained with REVERT total protein stain (Licor, USA) and detected using an Odyssey CLx scanner (Licor, USA) with the 700 nm channel. REVERT stain was then removed and RNaseJ1-6xHis protein was immunodetected with an anti-histidine antibody conjugated with CF680 dye (Sigma-Aldrich, Merck KGaA, Germany). RNaseJ1-6xHis protein was detected using the same 700 nm channel. Image Studio software (Licor, USA) was used for image capture; all unmodified images are shown in Supplementary data S2.

### Data analysis

R. 3.6.1 [56] was used for all data analyses. Data of cell turbidities (OD_600_) were analyzed using the grofit package [57]. Data from the individual experiments were fitted to model-free spline fits. The integral.spline values reported by grofit were used as growth values for analysis. Growth values of *E. coli* DH5α transformed with pBAD33 and pBAD33-rnjA-6xHis were fitted to linear regression models in R. Growth curves were generated using the growthcurve package [58]. Two sample, two-tailed Welch’s *t*-tests comparing growth between -ara and + ara condition were performed in R. Normalized fluorescence intensities were analysed by single value two-tailed *t*-tests comparing group mean to 1 in R. Statistical test *P*-values less than 0.05 were considered significant.

## Supplementary Information

**Additional file 1: Figure S1.** ffect of arabinose in plasmid transformed cells. Growth analysis of *E. coli* DH5α transformant carrying pBAD33 or pBAD33-rnjA-6xHis (pRJ1) treated with arabinose at varying concentrations. Points represent mean of 2–6 experiments and error bars represent SEM. Lines represent linear regression models to the data (*P* = 0.5261 and *P* = 0.0045 for pBAD33 and pRJ1, respectively).

**Additional file 2: Figure S2.** PCR results for the verification of gene integration at *lacZ* gene loci (**A**) and reporter plasmid transformation **(B).** iRJ1 and iBAD33 are abbreviations of *ΔlacZ*::RJ1, *ΔlacZ*::33, respectively. pRSETC_iRJ1, pEGFP-hDHFR_iRJ1, pRzI_iRJ1, and pRzII_iRJ1 are abbreviations of iRJ1 integrant transformed with pRSETC, pEGFP-hDHFR, pRzIEGFP-hDHFR, and pRzIIEGFP-hDHFR, respectively. gDNA from wild-type *E. coli* BL21(DE3) was used as a negative control. Primer flklacZ_F2 and AraC_R were used in PCR analysis in (A) and primer T7P_F and T7T_R were used in PCR analysis in (B). PCR products were separated in 0.8% agarose gel and stained with ethidium bromide. M indicates GeneRuler 1 kb DNA Ladder (Thermo Scientific, USA) and GeneRuler 1 kb Plus DNA Ladder (Thermo Scientific, USA) in (A) and (B), respectively.

**Additional file 3: Figure S3.** PCR results for the verification of gene integration at *arsB* (A) and *lacZ* (B) gene loci. iRzI_iRJ1, iRzI_i33, iRzII_iRJ1, iRzII_i33, iEGFP-hDHFR_iRJ1, iEGFP-hDHFR_i33, iRSETC_iRJ1, iRSETC_i33, i*glmS*_iRJ1, i*glmS*_i33, iM9_RJ1, and iM9_33 are abbreviations of double integrants of pBAD33 or at *arsB* gene locus and reporter plasmid pRSETC, pEGFP-hDHFR, pRzIEGFP-hDHFR, pRzIIEGFP-hDHFR, p*glmS*EGFP-hDHFR, or pM9EGFP-hDHFR at *lacZ* gene locus. gDNA from wild-type *E. coli* BL21(DE3) was used as a negative control in (A) and *ΔarsB*::RzIEGFP-hDHFR (iRzI single integrant) in (B). Primer flkarsB_F and T7T_R were used in PCR analysis in (A) and primer T7P_F and T7T_R were used in PCR analysis in (B). PCR products were separated in 0.8% agarose gel and stained with ethidium bromide. M indicates GeneRuler 1 kb Plus DNA Ladder (Thermo Scientific, USA).

**Additional file 4: Figure S4.** Growth analysis and fluorescence intensity of iBAD33 double integrants. (A) Growth analysis of double integrants; *ΔarsB*::EGFP-hDHFR*ΔlacZ*::33 (iEGFP-hDHFR_i33), *ΔarsB*::RzIEGFP-hDHFR*ΔlacZ*::33 (iRzI_i33), *ΔarsB*::RzIIEGFP-hDHFR*ΔlacZ*::33 (iRzII_i33), *ΔarsB*::*glmS*EGFP-hDHFR*ΔlacZ*::33 (i*glmS*_i33), and *ΔarsB*::M9EGFP-hDHFR*ΔlacZ*::33 (iM9_i33). OD_600_ was measured every hour from 0 to 8 h cultivation time and data were plotted using the Growthcurve package in R software. Data are shown for each cell type grown in the presence or absence of 0.0012% (w/v) arabinose. Points represent the mean of 4–12 experiments and error bars represent 95% confidence intervals. (B) Fluorescence intensity of i33 double integrants cultured in 0.0012% (w/v) arabinose relative to untreated control. Box plots show relative fluorescence data distribution. Dots indicate relative fluorescence data from individual experiments, and median values are indicated by the bold black line. *P-*values from single value two-tailed *t*-tests comparing group mean to 1 of EGFP-hDHFR_33, RzI_33, RzII_33, *glmS*_33, and M9_33 are 0.8902, 0.9995, 0.9644, 0.8562, and 0.8483 respectively.

**Additional file 5: Figure S5.** Western blot analysis of RNaseJ1-6xHis protein in single and double integrants All protein samples were extracted from integrants cultured in the absence (lane 1–3, 7–9, and 13–17) or presence of 0.0012% (w/v) arabinose (lane 4–6, 10–12, and 18–22) harvested after 8 h induction time. (A and C) Total protein-stained membrane (B and D) Immunodetection of RNaseJ1-6xHis using Anti-6X His IgG, CF™680 (Sigma-Aldrich, Merck KGaA, Germany). Lane 1 and 4 indicates protein lysates from pEGFP-hDHFR_iRJ1, lane 2 and 5 indicates protein lysates from pRzI_iRJ1, lane 3 and 6 indicates protein lysates from pRzII_iRJ1, lane 7, 10, 13 and 18 indicates protein lysates from iEGFP-hDHFR_iRJ1, lane 8, 11, 16 and 21 indicates protein lysates from iRzI_iRJ1, lane 9, 12, 17 and 22 indicates protein lysates from iRzII_iRJ1, lane 14 and 19 indicates protein lysates from i*glmS*_iRJ1, lane 15 and 20 indicates protein lysates from iM9_iRJ1, respectively. Lane M indicates Kaledioscope™ prestained protein ladder (Bio-Rad). Expected size of RNaseJ1-6xHis protein is 63 kDa.

**Additional file 6: Table S1.** List of oligo primers for construction of template and reporter plasmids. **Table S2.** List of oligo primers for preparation of DNA fragment for chromosome integration and verification of the integrants. **Table S3**. Growth and EGFP fluorescence of HH-co-transformants, HH-iRJ1single and double integrants detected at 6-h cultivation time.

**Additional file 7: Supplementary data S1.** Sequences of plasmids and DNA fragments for integration.

**Additional file 8: Supplementary data S2.** Unmodified images of gels and blots. Unmodified images are shown for each figure as indicated. The cropped regions are indicated by red boxes. Lanes with no labels outside of the red boxes indicate data unrelated to this study.

## Data Availability

All data generated or analysed during this study are included in this published article [and its supplementary information files].

## Abbreviations

6xHis: Six histidine residue tag
*arsB*: Gene coding for arsenite transporter
CMR: Chloramphenicol resistance gene
EGFP: Enhanced green fluorescent protein
GlcN6P: Glucosamine-6-phosphate
Ha: Hemagglutinin
HA: Homology arm
HDAG: Hi-def Azure supplemented with 0.5% glycerol
hDHFR: Human dihydrofolate reductase protein
KMR: Kanamycin resistance gene
*lacZ*: Gene encoding for beta-galactosidase
LB: Luria Bertani
M9CA: M9 minimal medium supplemented with 0.2% casamino acids
mRNA: Messenger RNA
nt: Nucleotide
OD_600_: Optical density at 600 nm
PCR: Polymerase chain reaction
PVDF: Polyvinylidene fluoride
RNA: Ribonucleic acid
rnjA: Gene encoding for *Bacillus subtilis* RNaseJ1
rRNA: Ribosomal RNA
SEM: Standard error of mean
