# Supplementary figures and images for: A cell-based ribozyme reporter system employing a chromosomally-integrated 5′ exonuclease gene
Source: BMC Mol Cell Biol. 2021 Mar 16;22:20. doi: 10.1186/s12860-021-00357-7 (PMC7967978; doi:10.1186/s12860-021-00357-7)

## Slide 1
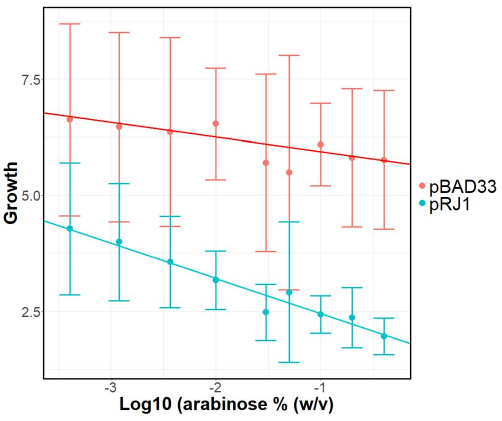

Supplement: Supplementary file 1 — Additional file 1: Figure S1. ffect of arabinose in plasmid transformed cells. Growth analysis of E. coli DH5α transformant carrying pBAD33 or pBAD33-rnjA-6xHis (pRJ1) treated with arabinose at varying concentrations. Points represent mean of 2–6 experiments and error bars represent SEM. Lines represent linear regression models to the data (P = 0.5261 and P = 0.0045 for pBAD33 and pRJ1, respectively). [file 12860_2021_357_MOESM1_ESM.pptx]

## Slide 1
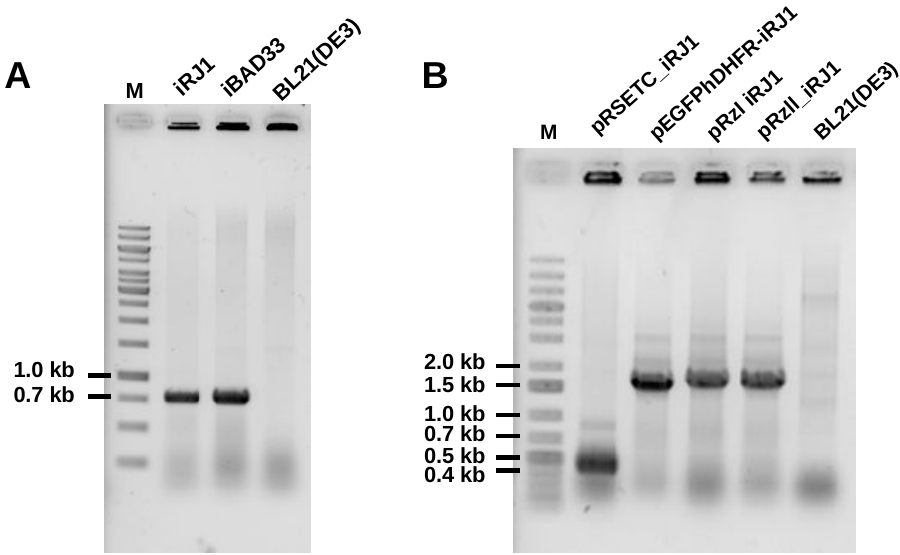

iRJ1
BL21(DE3)
iBAD33
A
B
pEGFPhDHFR-iRJ1
pRSETC_iRJ1
pRzI iRJ1
M
pRzll_iRJ1
BL21(DE3)
M
2.0 kb
1.0 kb
1.5 kb
0.7 kb
1.0 kb
0.7 kb
0.5 kb
0.4 kb

Supplement: Supplementary file 2 — Additional file 2: Figure S2. PCR results for the verification of gene integration at lacZ gene loci (A) and reporter plasmid transformation (B). iRJ1 and iBAD33 are abbreviations of ΔlacZ::RJ1, ΔlacZ::33, respectively. pRSETC_iRJ1, pEGFP-hDHFR_iRJ1, pRzI_iRJ1, and pRzII_iRJ1 are abbreviations of iRJ1 integrant transformed with pRSETC, pEGFP-hDHFR, pRzIEGFP-hDHFR, and pRzIIEGFP-hDHFR, respectively. gDNA from wild-type E. coli BL21(DE3) was used as a negative control. Primer flklacZ_F2 and AraC_R were used in PCR analysis in (A) and primer T7P_F and T7T_R were used in PCR analysis in (B). PCR products were separated in 0.8% agarose gel and stained with ethidium bromide. M indicates GeneRuler 1 kb DNA Ladder (Thermo Scientific, USA) and GeneRuler 1 kb Plus DNA Ladder (Thermo Scientific, USA) in (A) and (B), respectively. [file 12860_2021_357_MOESM2_ESM.pptx]

## Slide 1
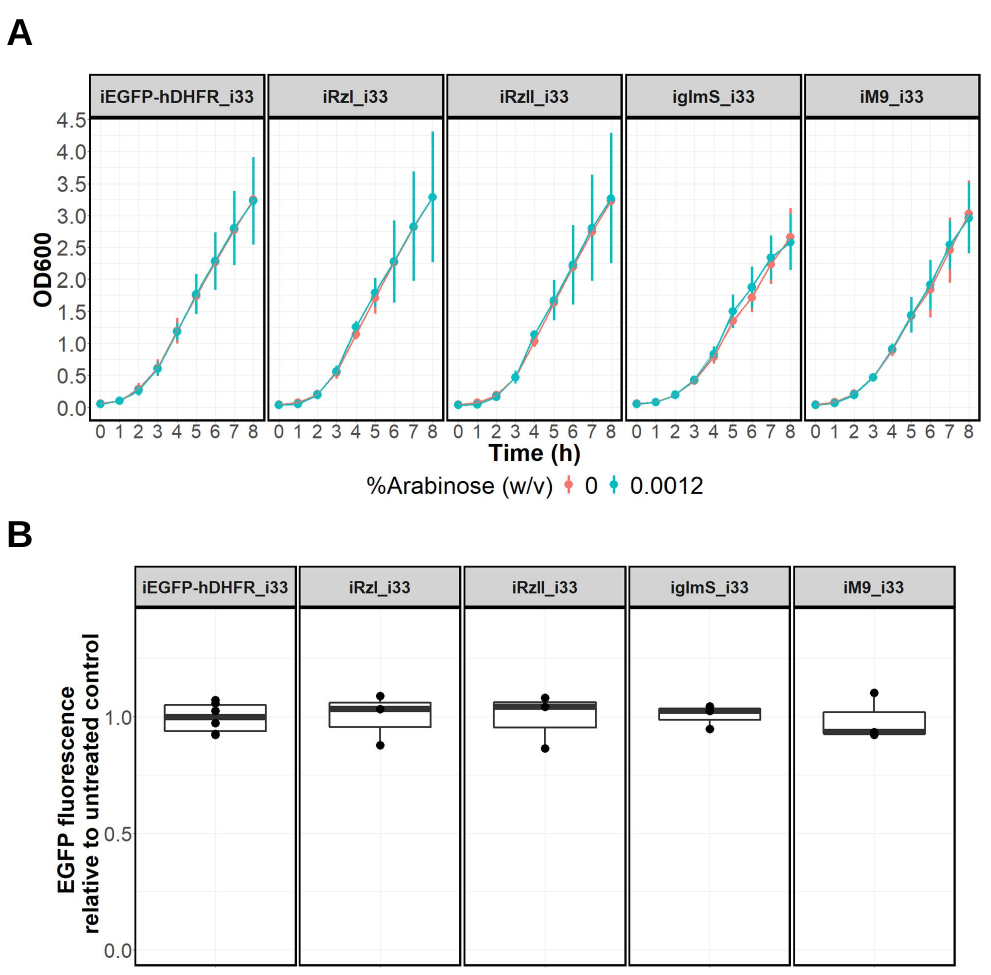

A
B

Supplement: Supplementary file 4 — Additional file 4: Figure S4. Growth analysis and fluorescence intensity of iBAD33 double integrants. (A) Growth analysis of double integrants; ΔarsB::EGFP-hDHFRΔlacZ::33 (iEGFP-hDHFR_i33), ΔarsB::RzIEGFP-hDHFRΔlacZ::33 (iRzI_i33), ΔarsB::RzIIEGFP-hDHFRΔlacZ::33 (iRzII_i33), ΔarsB::glmSEGFP-hDHFRΔlacZ::33 (iglmS_i33), and ΔarsB::M9EGFP-hDHFRΔlacZ::33 (iM9_i33). OD600 was measured every hour from 0 to 8 h cultivation time and data were plotted using the Growthcurve package in R software. Data are shown for each cell type grown in the presence or absence of 0.0012% (w/v) arabinose. Points represent the mean of 4–12 experiments and error bars represent 95% confidence intervals. (B) Fluorescence intensity of i33 double integrants cultured in 0.0012% (w/v) arabinose relative to untreated control. Box plots show relative fluorescence data distribution. Dots indicate relative fluorescence data from individual experiments, and median values are indicated by the bold black line. P-values from single value two-tailed t-tests comparing group mean to 1 of EGFP-hDHFR_33, RzI_33, RzII_33, glmS_33, and M9_33 are 0.8902, 0.9995, 0.9644, 0.8562, and 0.8483 respectively. [file 12860_2021_357_MOESM4_ESM.pptx]

## Slide 1
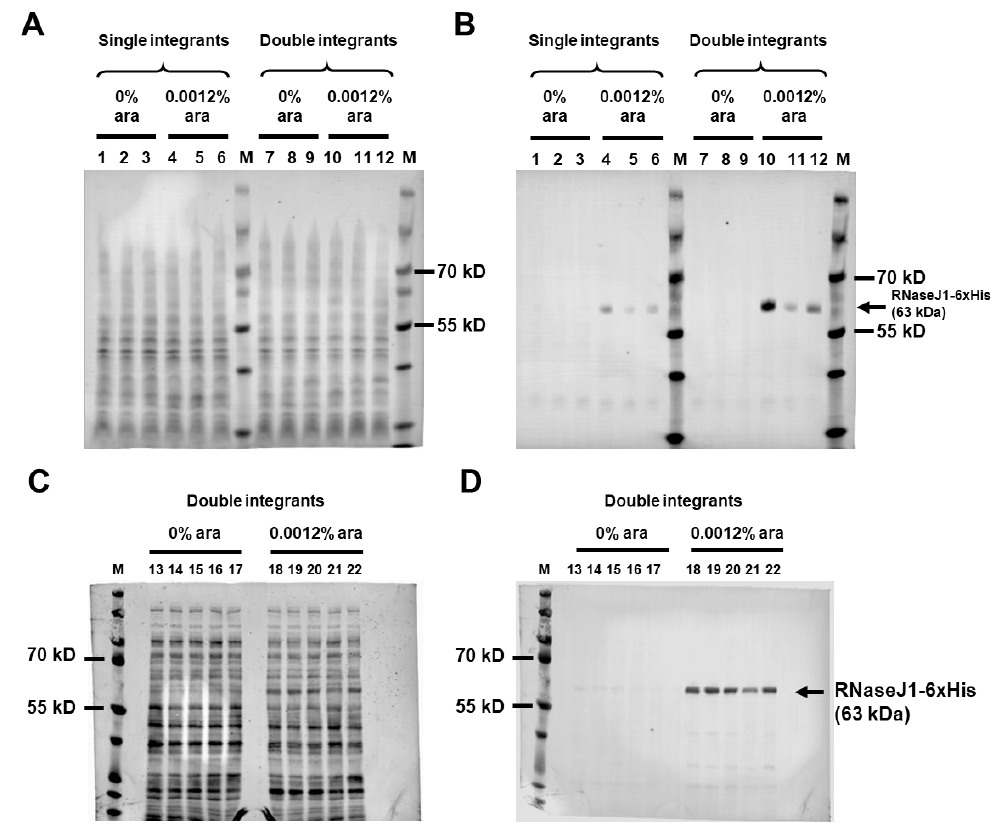

Supplement: Supplementary file 5 — Additional file 5: Figure S5. Western blot analysis of RNaseJ1-6xHis protein in single and double integrants All protein samples were extracted from integrants cultured in the absence (lane 1–3, 7–9, and 13–17) or presence of 0.0012% (w/v) arabinose (lane 4–6, 10–12, and 18–22) harvested after 8 h induction time. (A and C) Total protein-stained membrane (B and D) Immunodetection of RNaseJ1-6xHis using Anti-6X His IgG, CF™680 (Sigma-Aldrich, Merck KGaA, Germany). Lane 1 and 4 indicates protein lysates from pEGFP-hDHFR_iRJ1, lane 2 and 5 indicates protein lysates from pRzI_iRJ1, lane 3 and 6 indicates protein lysates from pRzII_iRJ1, lane 7, 10, 13 and 18 indicates protein lysates from iEGFP-hDHFR_iRJ1, lane 8, 11, 16 and 21 indicates protein lysates from iRzI_iRJ1, lane 9, 12, 17 and 22 indicates protein lysates from iRzII_iRJ1, lane 14 and 19 indicates protein lysates from iglmS_iRJ1, lane 15 and 20 indicates protein lysates from iM9_iRJ1, respectively. Lane M indicates Kaledioscope™ prestained protein ladder (Bio-Rad). Expected size of RNaseJ1-6xHis protein is 63 kDa. [file 12860_2021_357_MOESM5_ESM.pptx]
