# Supplementary material for: A cell-based ribozyme reporter system employing a chromosomally-integrated 5′ exonuclease gene
Source: BMC Mol Cell Biol. 2021 Mar 16;22:20. doi: 10.1186/s12860-021-00357-7 (PMC7967978; doi:10.1186/s12860-021-00357-7)
Supplement: Supplementary file 3 — Additional file 3: Figure S3. PCR results for the verification of gene integration at arsB (A) and lacZ (B) gene loci. iRzI_iRJ1, iRzI_i33, iRzII_iRJ1, iRzII_i33, iEGFP-hDHFR_iRJ1, iEGFP-hDHFR_i33, iRSETC_iRJ1, iRSETC_i33, iglmS_iRJ1, iglmS_i33, iM9_RJ1, and iM9_33 are abbreviations of double integrants of pBAD33 or at arsB gene locus and reporter plasmid pRSETC, pEGFP-hDHFR, pRzIEGFP-hDHFR, pRzIIEGFP-hDHFR, pglmSEGFP-hDHFR, or pM9EGFP-hDHFR at lacZ gene locus. gDNA from wild-type E. coli BL21(DE3) was used as a negative control in (A) and ΔarsB::RzIEGFP-hDHFR (iRzI single integrant) in (B). Primer flkarsB_F and T7T_R were used in PCR analysis in (A) and primer T7P_F and T7T_R were used in PCR analysis in (B). PCR products were separated in 0.8% agarose gel and stained with ethidium bromide. M indicates GeneRuler 1 kb Plus DNA Ladder (Thermo Scientific, USA). [file 12860_2021_357_MOESM3_ESM.pptx]

## Slide 1
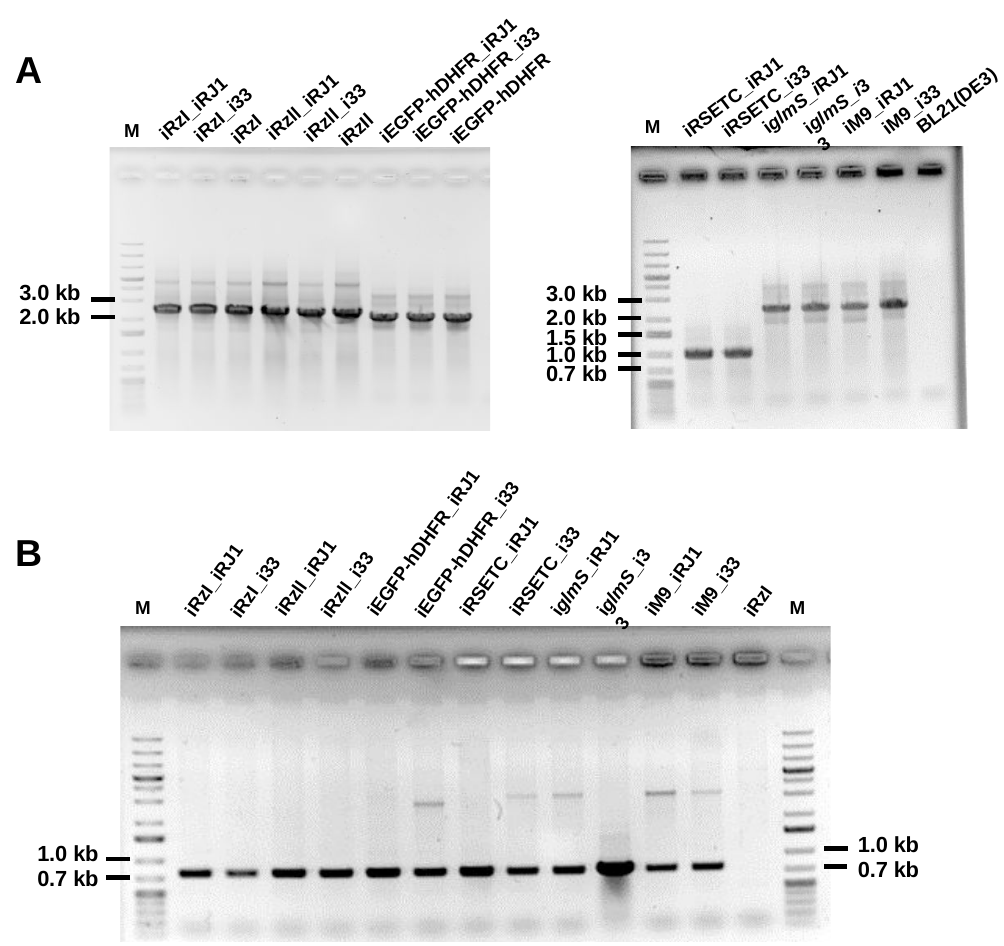

A
iEGFP-hDHFR_i33
iRzIl
iEGFP-hDHFR_iRJ1
iRzI
iRzll_i33
iRzll_iRJ1
iRSETC_i33
iEGFP-hDHFR
iRSETC_iRJ1
iglmS_iRJ1
iRzI_iRJ1
BL21(DE3)
iRzI_i33
iM9_iRJ1
iglmS_i33
iM9_i33
M
M
3.0 kb
3.0 kb
2.0 kb
2.0 kb
1.5 kb
1.0 kb
0.7 kb
iRzI
iEGFP-hDHFR_iRJ1
iEGFP-hDHFR_i33
B
iRSETC_iRJ1
iglmS_iRJ1
iRSETC_i33
iM9_iRJ1
iRzll_iRJ1
iRzI_iRJ1
iglmS_i33
iM9_i33
iRzll_i33
iRzI_i33
M
M
1.0 kb
1.0 kb
0.7 kb
0.7 kb
