# Supplementary material for: A cell-based ribozyme reporter system employing a chromosomally-integrated 5′ exonuclease gene
Source: BMC Mol Cell Biol. 2021 Mar 16;22:20. doi: 10.1186/s12860-021-00357-7 (PMC7967978; doi:10.1186/s12860-021-00357-7)
Supplement: Supplementary file 6 — Additional file 6: Table S1. List of oligo primers for construction of template and reporter plasmids. Table S2. List of oligo primers for preparation of DNA fragment for chromosome integration and verification of the integrants. Table S3. Growth and EGFP fluorescence of HH-co-transformants, HH-iRJ1single and double integrants detected at 6-h cultivation time. [file 12860_2021_357_MOESM6_ESM.docx]

**Table S1 List of oligo primers for construction of template and reporter plasmids**

*The underlined sequences indicate restriction enzyme site shown in “Restriction enzyme” column. Bold sequences indicate a mutated nucleotide in the RzII inactive hammer head ribozyme (Zhang, Stancek, and Isaksson 1997).

| **Oligo primers** | **Sequence (5′-3′)*** | **Restriction enzyme** |
| --- | --- | --- |
| HaEGFP_F | GGAGGAAGAAAAATATGGCATACCCTTATGACGTACCTGACTA |  |
| HaEGFP_R | CGGGGTACCGCTACCTCCGCCACCACTTCCACCGCCTCCAGAACCTCCTCCACCCTTGTACAGCTCGTCCATG | KpnI |
| hDHFR_F | CGGGGTACCATGGTTGGTTCGCTAAACTG | KpnI |
| hDHFRR4 | AAAACTGCAGTTAATCATTCTTCTCATATA CTTCAAATTTG | PstI |
| EGFPhDHFR_F | CGCGGATCCAAAAATA TGGCATACCCTTATGA | BamHI |
| EGFPhDHFR_R | ACCGCTCGAGTTAATCATTCTTCTCATATACTTCA AATTTG | XhoI |
| EGFP_F | AAAACATATGAGCAAGGGCGAGGAGCTGTT | NdeI |
| BsglmS_F | TATAATTATAGCGCCCGAACTAA |  |
| 168BsglmS_R | AGATCATGTGATTTCTCTTT |  |
| pBEAR166*glmS*gibson_F | ATAGGGAGACCACAACGGTTTCCCTGGATCCTAATTAT**AG**CGCCCGAACTAA | BamHI |
| pBEAR166M9gibson_F | ATAGGGAGACCACAACGGTTTCCCTGGATCCTAATTAT**CC**CGCCCGAACTAA | BamHI |
| pBEAR166*glmS*gibson_R | TAAAGTTAAACAAAATTATTTCTAGGATATCAGATCATGTGATTTCTCTTTGTT | EcoRV |
| hDHFRgap_F | CTAGAAATAATTTTGTTTAACTTTAAGAAG |  |
| hDHFRgap_R | TTCAGTAAGTTTTAAGGCATCATCTA |  |
| RzI_F | GAGACCACAACGGTTTCCCTGGATCCGCCTGTCACCGGATGTGTTTTCCGGTCTGATGAGTCCGT | - |
| RzI_R | GTTAAACAAAATTATTTCTAGGATTCCTGTTTCGTCCTCACGGACTCATCAGACCGGAAA | - |
| RzII_F | GAGACCACAACGGTTTCCCTGGATCCGCCTGT**G**ACCGGATGTGTTTTCCGGTCTGATGAGTCCGT | - |

**Table S2 List of oligo primers for preparation of DNA fragment for chromosome integration and verification of the integrants**

*The underlined sequences indicate restriction enzyme site shown in “Restriction enzyme” column.

| **Oligo primers** | **Sequence (5′-3′)*** | **Restriction enzyme** | **PCR amplification** | **DNA template** | **Amplicon size (bp)** |
| --- | --- | --- | --- | --- | --- |
| HA1arsB_F | CCGGCTCGAGATGTTACTGGCAGGCGCTATCTTTG | XhoI | 5′homology arm of *arsB* gene fragment | gDNA of *E. coli* BL21(DE3) | 535 |
| HA1arsB_R | GTATTAATTTCGCGGGATCGAGATCAAATCAGCGGAAACGATATTCACCA | - |  |  |  |
| HA2arsB_F | ACTTCGGAATAGGAACTAAGGAGGAAACGCGGTCATGCGATTAATACGGG | - | 3′homology arm of *arsB* gene fragment | gDNA of *E. coli* BL21(DE3) | 535 |
| HA2arsB_R | ATAGGCTAGCTTACAAAGTGAAAGAGAGACGTAGCGC | NheI |  |  |  |
| FRT_F | GTGTAGGCTGGAGCTGCTTCGAAGT | - | FRT-kanamycin fragment | pKD4 plasmid | 1,467 |
| FRT_R | CTTTACCCGTATTAATCGCATGACCGCGTTTCCTCCTTAGTTCCTATTCCGAAGT | - |  |  |  |
| T7P_F | CAACCTGGTGAATATCGTTTCCGCTGATTTGATCTCGATCCCGCGAAATTAATAC | - | Reporter gene fragment | RSETC plasmid  noRz plasmid  *glmS* plasmid  M9 plasmid  RzI plasmid  RzII plasmid | 377  1,644  1,822  1,822  1,699  1,699 |
| T7T_R | ACTTCGAAGCAGCTCCAGCCTACACGATATAGTTCCTCCTTTCAGCAAAAAACCC | - |  |  |  |
| HA1lacZ_F | CCGGCTCGAGTTATTTTTGACACCAGACCAACTGG | XhoI | 5′homology arm of *lacZ* gene fragment | gDNA of *E. coli* BL21(DE3) | 535 |
| lacZAraC_R | AACGAATCAGACAATTGACGGCTTGTTGATGGTAGTGGTCAAATGGCGAT | - |  |  |  |
| HA2lacZ_F | GATCAACGTCTCATTTTCGCCAAAACGACTGTCCTGGCCGTAACCGACCC | - | 3′homology arm of *lacZ* gene fragment | gDNA of *E. coli* BL21(DE3) | 535 |
| HA2lacZ_R | CTAGTCTAGAATGACCATGATTACGGATTCACTGG | XbaI |  |  |  |
| AraC_F | CGGTAATCGCCATTTGACCACTACCATCAACAAGCCGTCAATTGTCTGATTCGTT | - | araC-rnjA(BAD33)-chloramphenicol fragment | pBAD33 plasmid  pBAD33::rnjA | 4,121  5,799 |
| RJ1_R | GCGCTGGGTCGGTTACGGCCAGGACAGTCGTTTTGGCGAAAATGAGACGTTGATC | - |  |  |  |
| flkarsB_F | GGACAGTAAGAACATTTGCAGTT | - | Verification of *ΔarsB* integrant with primer T7T_R | gDNA of the *ΔarsB* integrant  RSETC integrant  noRz integrant  *glmS* integrant  M9 integrant  RzI integrant  RzII integrant | 980  2,192  2,370  2,370  2,247  2,247 |
| flklacZ_F2 | TACGCGAAATACGGGCAGACA | - | Verification of *ΔlacZ* integrant | gDNA of the *ΔlacZ* integrant | 770 |
| AraC_R | CGCGAGGACCAACGTATCAG | - |  |  |  |

**Table S3** **Growth and EGFP fluorescence of HH-co-transformants, HH-iRJ1single and double integrants detected at 6-h cultivation time**

| Strains | Replicates | Arabinose (% w/v) | Average OD600 | Average fluorescence intensity | Normalized fluorescence intensity | Relative %fluorescence intensity to arabinose un-treated control |
| --- | --- | --- | --- | --- | --- | --- |
| pRzI_p33 | 1 | 0 | 0.61 | 13670.33 | 22312.84 | 100 |
|  |  | 0.0012 | 0.68 | 13156.67 | 19343.30 | 87 |
|  | 2 | 0 | -0.02 | 16609.33 | -958230.77 | 100 |
|  |  | 0.0012 | 0.00 | 16922.67 | -20307200.00 | 2119 |
|  | 3 | 0 | 0.01 | 19783.33 | 3297222.22 | 100 |
|  |  | 0.0012 | 0.01 | 20027.33 | 2071793.10 | 63 |
|  | 4 | 0 | 0.12 | 9888.00 | 83560.56 | 100 |
|  |  | 0.0012 | 0.12 | 9940.67 | 82041.27 | 98 |
| pRzI_pRJ1 | 1 | 0 | 3.17 | 945.33 | 297.95 | 100 |
|  |  | 0.0012 | 2.45 | 500.67 | 203.99 | 68 |
|  | 2 | 0 | -0.17 | 72.33 | -424.66 | 100 |
|  |  | 0.0012 | -0.16 | 58.33 | -372.74 | 88 |
|  | 3 | 0 | 2.48 | 3926.67 | 1583.55 | 100 |
|  |  | 0.0012 | 1.92 | 3937.00 | 2046.44 | 129 |
|  | 4 | 0 | 2.48 | 1679.00 | 678.29 | 100 |
|  |  | 0.0012 | 1.81 | 1622.00 | 897.04 | 132 |
| pRzII_p33 | 1 | 0 | 2.71 | 2586.67 | 956.25 | 100 |
|  |  | 0.0012 | 2.88 | 2513.00 | 872.52 | 91 |
|  | 2 | 0 | 0.17 | 12924.00 | 75212.42 | 100 |
|  |  | 0.0012 | 0.22 | 12828.67 | 57830.20 | 77 |
|  | 3 | 0 | 3.26 | 2321.67 | 711.48 | 100 |
|  |  | 0.0012 | 3.35 | 2297.00 | 685.47 | 96 |
|  | 4 | 0 | 3.06 | 4117.67 | 1346.45 | 100 |
|  |  | 0.0012 | 3.11 | 4019.67 | 1291.32 | 96 |
| pRzII_pRJ1 | 1 | 0 | 2.23 | 2083.67 | 936.34 | 100 |
|  |  | 0.0012 | 2.13 | 1901.67 | 892.87 | 95 |
|  | 2 | 0 | 2.91 | 3814.00 | 1310.88 | 100 |
|  |  | 0.0012 | 2.48 | 3889.00 | 1568.04 | 120 |
|  | 3 | 0 | 0.02 | 15711.67 | 992315.79 | 100 |
|  |  | 0.0012 | 0.02 | 15297.67 | 891126.21 | 90 |
|  | 4 | 0 | 0.09 | 7556.67 | 83653.14 | 100 |
|  |  | 0.0012 | 0.10 | 7638.33 | 78881.24 | 94 |
| pRzI_iRJ1 | 1 | 0 | 2.91 | 887.00 | 304.57 | 100 |
|  |  | 0.0012 | 2.35 | 540.33 | 229.68 | 75 |
|  | 2 | 0 | 3.18 | 888.33 | 278.93 | 100 |
|  |  | 0.0012 | 2.41 | 527.67 | 218.86 | 78 |
|  | 3 | 0 | 3.18 | 1024.33 | 321.63 | 100 |
|  |  | 0.0012 | 2.41 | 624.67 | 259.09 | 81 |
|  | 4 | 0 | 3.18 | 819.67 | 257.37 | 100 |
|  |  | 0.0012 | 2.41 | 638.33 | 264.76 | 103 |
|  | 5 | 0 | 3.18 | 892.67 | 280.29 | 100 |
|  |  | 0.0012 | 2.41 | 600.00 | 248.86 | 89 |
| pRzII_iRJ1 | 1 | 0 | 3.14 | 884.00 | 281.91 | 100 |
|  |  | 0.0012 | 2.40 | 588.67 | 245.64 | 87 |
|  | 2 | 0 | 2.86 | 710.00 | 248.16 | 100 |
|  |  | 0.0012 | 2.42 | 612.67 | 253.45 | 102 |
|  | 3 | 0 | 2.86 | 1029.00 | 359.65 | 100 |
|  |  | 0.0012 | 2.42 | 593.67 | 245.59 | 68 |
|  | 4 | 0 | 2.86 | 829.00 | 289.75 | 100 |
|  |  | 0.0012 | 2.42 | 544.33 | 225.18 | 78 |
|  | 5 | 0 | 2.86 | 890.00 | 311.07 | 100 |
|  |  | 0.0012 | 2.42 | 603.00 | 249.45 | 80 |
| iRzI_iRJ1 | 1 | 0 | 1.947 | 3952.000 | 2029.789 | 100 |
|  |  | 0.0012 | 1.614 | 1683.000 | 1042.643 | 51 |
|  | 2 | 0 | 2.263 | 4115.333 | 1818.396 | 100 |
|  |  | 0.0012 | 1.901 | 1674.667 | 881.172 | 48 |
|  | 3 | 0 | 2.610 | 3973.000 | 1522.319 | 100 |
|  |  | 0.0012 | 2.481 | 1690.333 | 681.311 | 45 |
|  | 4 | 0 | 2.380 | 5763.667 | 2422.218 | 100 |
|  |  | 0.0012 | 2.109 | 2468.000 | 1170.500 | 48 |
|  | 5 | 0 | 1.944 | 3503.667 | 1802.143 | 100 |
|  |  | 0.0012 | 1.641 | 1643.333 | 1001.422 | 56 |
|  | 6 | 0 | 1.555 | 2466.333 | 1586.407 | 100 |
|  |  | 0.0012 | 1.417 | 1264.333 | 892.471 | 56 |
|  | 7 | 0 | 2.330 | 3508.000 | 1505.903 | 100 |
|  |  | 0.0012 | 2.110 | 1717.333 | 814.095 | 54 |
| iRzII_iRJ1 | 1 | 0 | 1.886 | 7980.667 | 4231.157 | 100 |
|  |  | 0.0012 | 1.376 | 7557.333 | 5491.583 | 130 |
|  | 2 | 0 | 2.193 | 8522.333 | 3885.858 | 100 |
|  |  | 0.0012 | 1.844 | 7355.000 | 3989.333 | 103 |
|  | 3 | 0 | 2.562 | 4319.000 | 1686.012 | 100 |
|  |  | 0.0012 | 2.376 | 4095.000 | 1723.727 | 102 |
|  | 4 | 0 | 2.258 | 6984.667 | 3092.841 | 100 |
|  |  | 0.0012 | 1.810 | 6651.000 | 3674.924 | 119 |
|  | 5 | 0 | 1.869 | 5621.667 | 3007.579 | 100 |
|  |  | 0.0012 | 1.720 | 5260.000 | 3057.547 | 102 |
|  | 6 | 0 | 1.621 | 5155.000 | 3180.136 | 100 |
|  |  | 0.0012 | 1.421 | 5067.000 | 3564.962 | 112 |
|  | 7 | 0 | 2.439 | 6679.000 | 2738.417 | 100 |
|  |  | 0.0012 | 1.990 | 6203.000 | 3116.824 | 114 |
| i*glmS*_iRJ1 | 1 | 0 | 2.221 | 424.333 | 191.041 | 100 |
|  |  | 0.0012 | 1.917 | 243.000 | 126.794 | 66 |
|  | 2 | 0 | 1.883 | 342.000 | 181.625 | 100 |
|  |  | 0.0012 | 1.751 | 201.000 | 114.781 | 63 |
|  | 3 | 0 | 1.612 | 329.667 | 204.508 | 100 |
|  |  | 0.0012 | 1.251 | 183.333 | 146.549 | 72 |
| iM9_iRJ1 | 1 | 0 | 2.132 | 1537.333 | 721.245 | 100 |
|  |  | 0.0012 | 1.759 | 1173.000 | 667.046 | 93 |
|  | 2 | 0 | 1.651 | 1238.667 | 750.404 | 100 |
|  |  | 0.0012 | 1.367 | 1000.000 | 731.440 | 98 |
|  | 3 | 0 | 1.721 | 1307.667 | 759.682 | 100 |
|  |  | 0.0012 | 1.530 | 1181.667 | 772.584 | 102 |
