# Supplementary material for: A cell-based ribozyme reporter system employing a chromosomally-integrated 5′ exonuclease gene
Source: BMC Mol Cell Biol. 2021 Mar 16;22:20. doi: 10.1186/s12860-021-00357-7 (PMC7967978; doi:10.1186/s12860-021-00357-7)

**Supplementary data S2 Unmodified images of gels and blots.** Unmodified images are shown for each figure as indicated. The cropped regions are indicated by red boxes. Lanes with no labels outside of the red boxes indicate data unrelated to this study.

1. Uncropped image for Fig. 2A


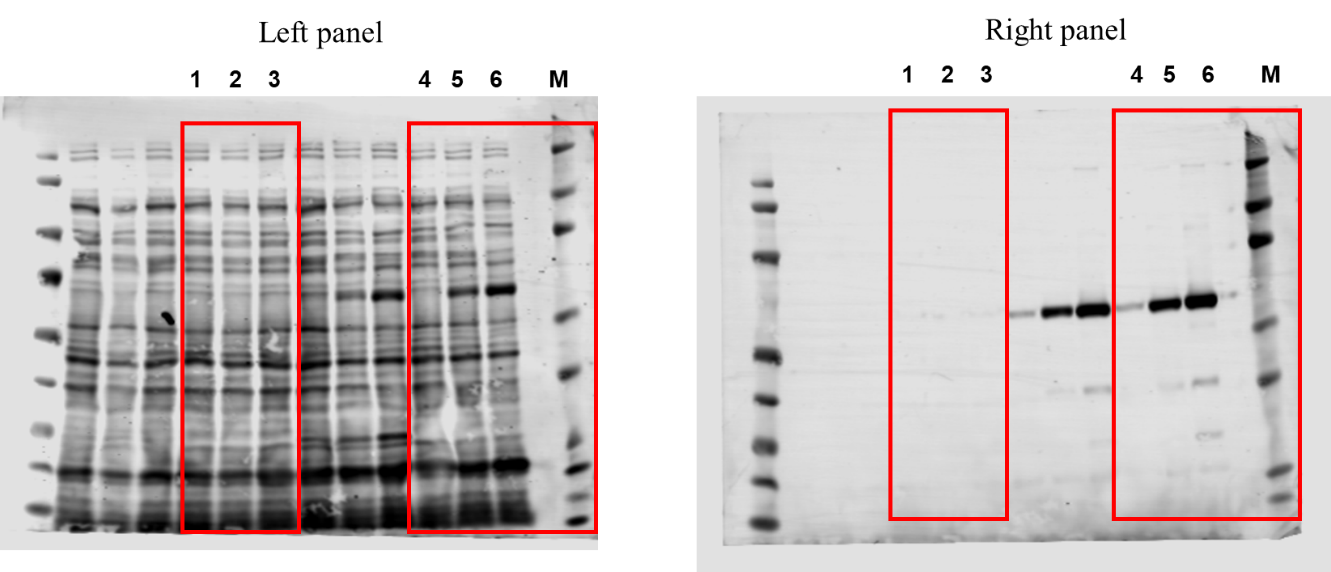


1. Uncropped image for Fig. 4A


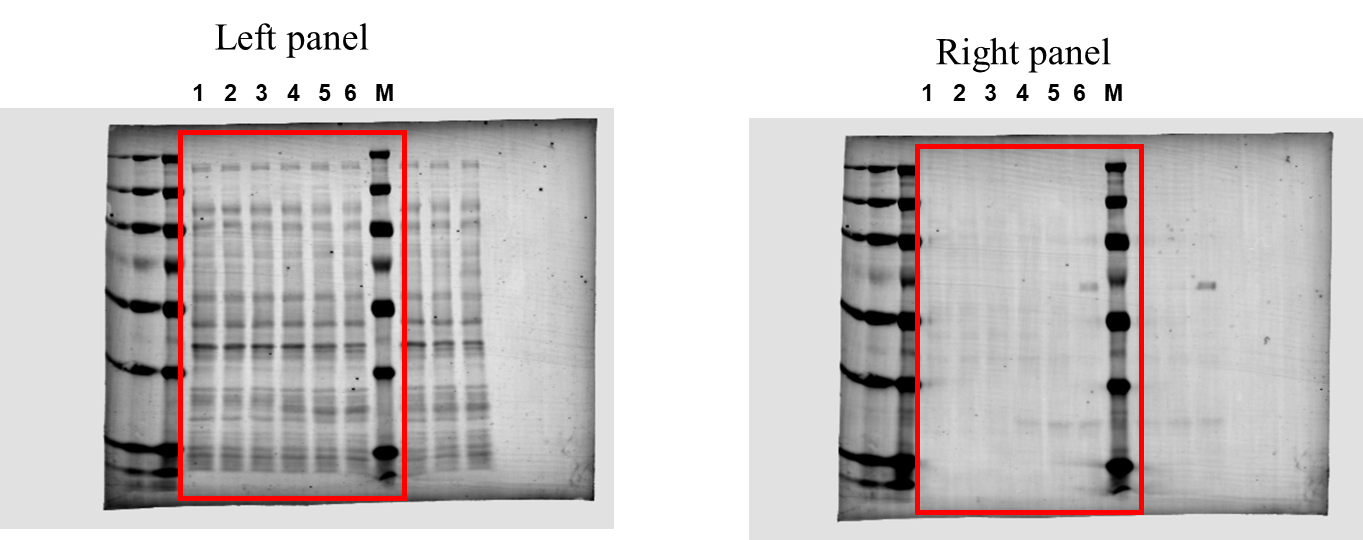


1. Uncropped image for Fig. S2A and S2B


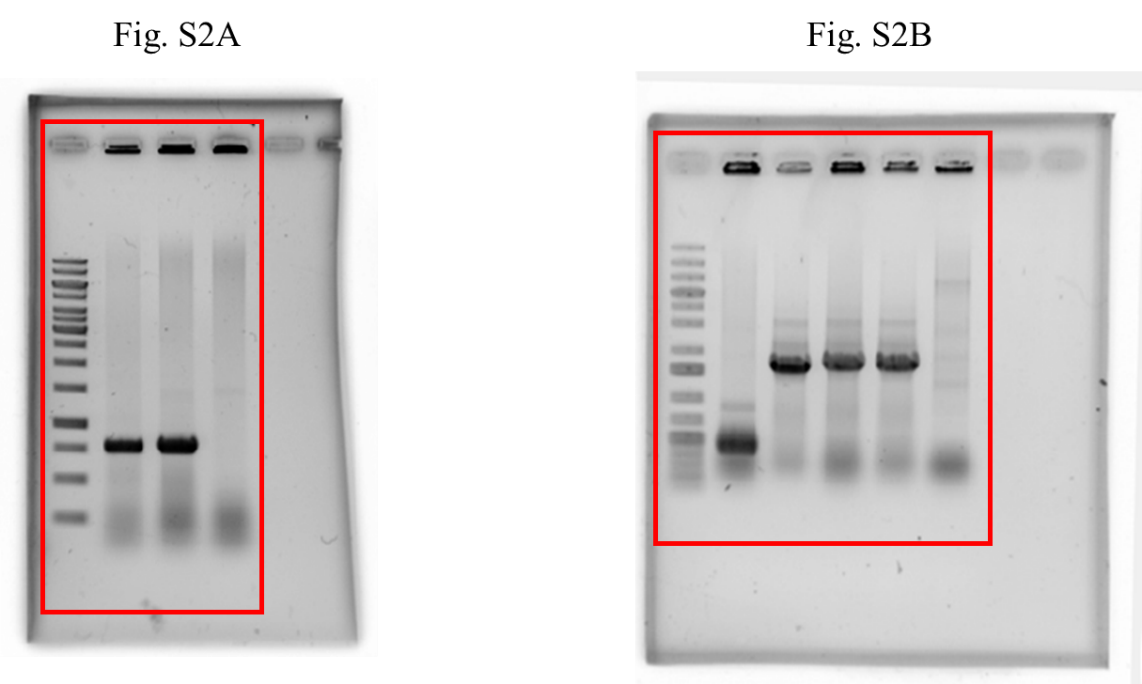


1. Uncropped image for S3A and S3B


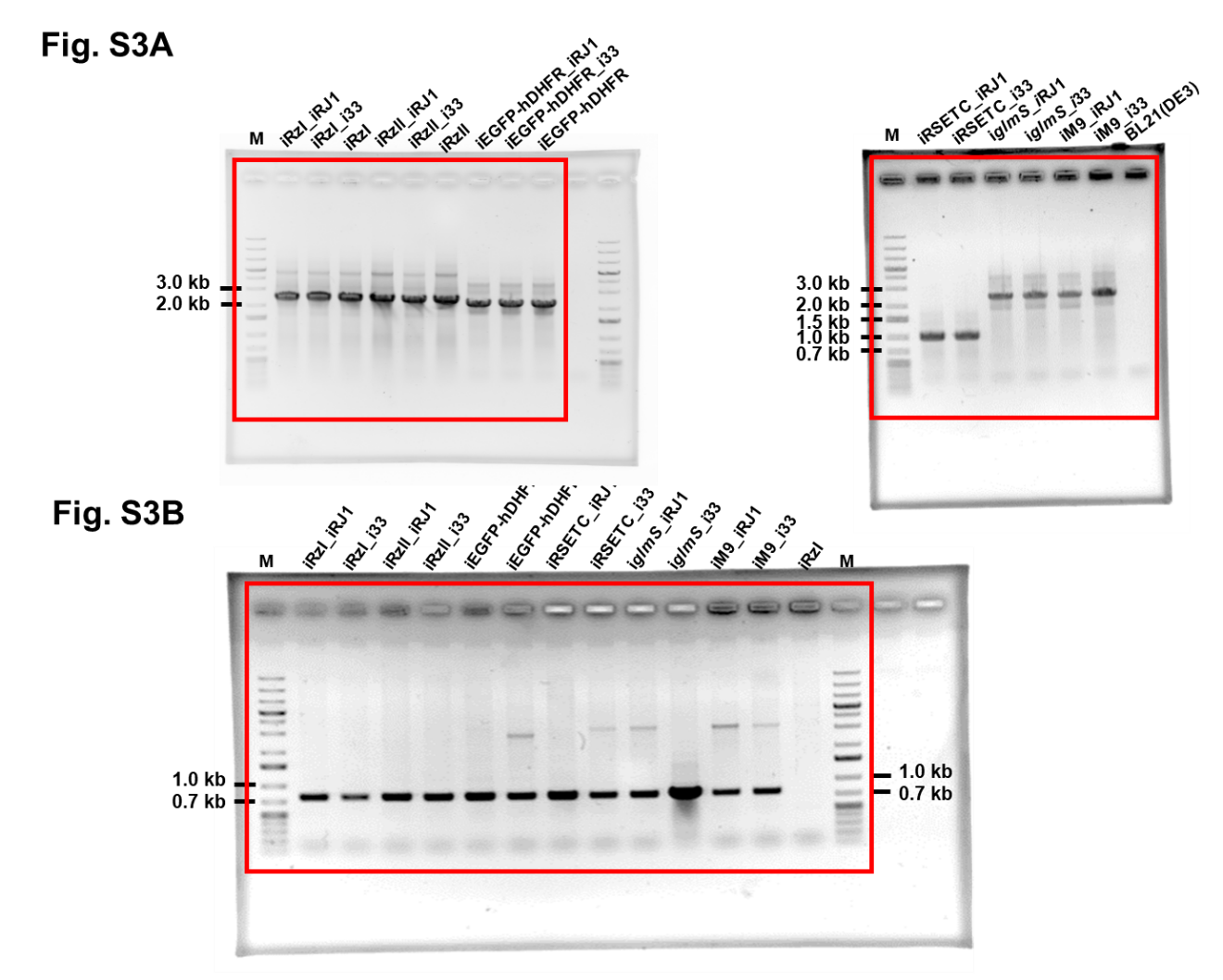


1. Uncropped image for S5A, S5B, S5C, and S5D


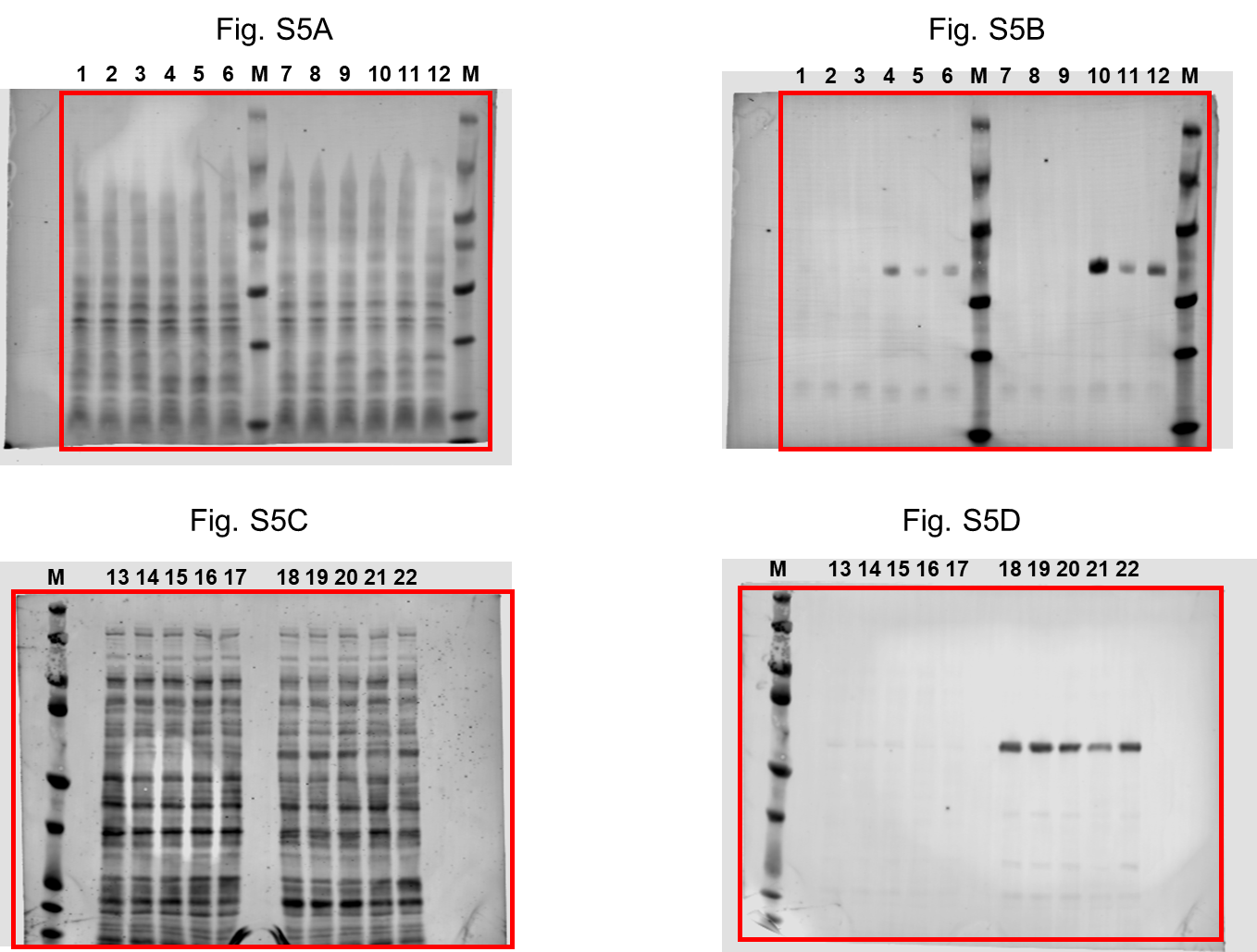

Supplement: Supplementary file 8 — Additional file 8: Supplementary data S2. Unmodified images of gels and blots. Unmodified images are shown for each figure as indicated. The cropped regions are indicated by red boxes. Lanes with no labels outside of the red boxes indicate data unrelated to this study. [file 12860_2021_357_MOESM8_ESM.docx]
